# Transition from Parenteral to Subcutaneous Application of Systemic Oncological Therapy for Treating Non-Small-Cell Lung Cancer

**DOI:** 10.3390/curroncol33060307

**Published:** 2026-05-25

**Authors:** Anela Muratovic, Urska Janzic

**Affiliations:** 1 Department of Medical Oncology, University Clinic Golnik, 4204 Golnik, Slovenia; anela.muratovic@klinika-golnik.si; 2 Medical Faculty, University of Ljubljana, 1000 Ljubljana, Slovenia

**Keywords:** lung cancer, oncological therapy, change management, innovations in healthcare, quality of medical treatment

## Abstract

Background: The transition from the parenteral to subcutaneous application of systemic oncological therapy represents one of the most important innovations in modern oncology, as it affects the quality of life of patients as well as the organization of work and the management of health services. The introduction of this change requires effective leadership, interdisciplinary cooperation, and the adaptation of existing processes in healthcare organizations. Methods: We conducted a systematic review of the professional and scientific literature, considering the purpose and goal of this research. We used electronic databases: Wiley Online Library, PubMed, COBBIS.SI, and Google Scholar web browser. Papers from 2017 to 2025 were considered and processed using meta-synthesis. Results: Recent studies confirm that the subcutaneous administration of immunotherapy and targeted therapy is as effective and safe as parenteral immunotherapy, while significantly reducing treatment time and improving patient experience. Discussion: The transition to subcutaneous application provides an opportunity to transform oncology care. From a management perspective, the introduction of subcutaneous application requires systematic change management, staff training, process adaptation, and interdisciplinary cooperation. The sustainable implementation of innovations depends on organization, communication, and management support.

## Simple Summary

The shift from intravenous to subcutaneous administration of systemic oncology treatments is an important innovation that improves patient experience and optimizes healthcare workflows. To understand its impact, we conducted a systematic review of the recent scientific and professional literature (2017–2025) using major medical databases. The evidence shows that subcutaneous oncological therapy is as equally effective and safe as intravenous treatment, significantly shortens treatment time, and enhances patient satisfaction. Successfully implementing this transition requires coordinated leadership, staff training, process adaptation, and strong interdisciplinary collaboration. The sustainable integration of subcutaneous administration into oncology practice depends on effective organization, communication, and managerial support.

## 1. Introduction

Lung cancer remains the leading cause of cancer-related mortality worldwide, accounting for more deaths than breast, colorectal and prostate cancer combined [1]. Despite improvements in screening, diagnostics and treatment, the majority of patients continue to present with advanced-stage disease, where systemic therapy is the cornerstone of management. Over the past decade, major advances in the molecular characterization of non-small-cell lung cancer (NSCLC) have enabled the development of targeted therapies and immune checkpoint inhibitors, fundamentally transforming treatment outcomes [2].

Immune checkpoint inhibitors, such as nivolumab, pembrolizumab and atezolizumab, as well as targeted agents including amivantamab and other next-generation inhibitors, have become standard of care for molecularly selected subgroups of patients with advanced NSCLC [3]. These therapies have demonstrated durable responses and survival benefits in multiple landmark trials. However, their integration into routine clinical practice has introduced substantial organizational and logistical challenges. Intravenous (IV) administration requires prolonged infusion times, dedicated nursing supervision, infusion pumps, monitoring equipment, and adequate space in day-hospital units. For many patients, this translates into long hospital visits, transportation burdens, and reduced flexibility in daily life. For healthcare systems, IV administration increases workload, resource consumption and operational complexity [2].

In recent years, the development of subcutaneous (SC) formulations of systemic oncologic therapies has emerged as a major innovation with the potential to reshape oncology care delivery. SC administration is a faster, simpler and more patient-centered alternative to IV infusion. Clinical trials have consistently shown that SC formulations of immunotherapy and targeted agents are non-inferior in efficacy and safety compared with their IV counterparts, while providing substantial organizational advantages [3,4]. SC administration significantly shortens treatment time—from hours to minutes—improves patient throughput, reduces chair time, and allows more efficient use of healthcare resources. From a quality-of-care perspective, SC delivery reduces the need for prolonged hospital stays, improves workflow fluidity, and enables healthcare professionals to allocate more time to patients requiring complex care [2,5].

Beyond clinical and operational benefits, the transition to SC administration provides broader opportunities for innovation and transformation in oncology practice. Implementing SC therapies requires coordinated change management, including staff training, adaptation of clinical pathways, reorganization of patient flow, and strong interdisciplinary collaboration. Successful and sustainable adoption depends on leadership engagement, clear communication, and the alignment of organizational processes with the new mode of delivery.

The purpose of this review is to provide a comprehensive overview of the benefits and challenges associated with transitioning from parenteral to subcutaneous administration of oncologic therapies. We examine the clinical evidence supporting SC formulations, analyze their impact on patient experience and healthcare system efficiency, and discuss the organizational and managerial considerations necessary for successful implementation. Finally, we highlight the gaps in current knowledge and propose directions for future research, including real-world evaluations of SC adoption, cost-effectiveness analyses, and patient-reported outcomes.

## 2. Methods

This research was carried out as a systematic review of the professional and scientific literature, considering the purpose and goal of this research. This analysis was based on a descriptive approach of the work by analyzing, comparing and summarizing the findings of the peer-reviewed literature. We focused on analyzing the scientific evidence on the efficacy and safety of subcutaneous therapy in the treatment of lung cancer, evaluating the organizational and personnel effects of the introduction of subcutaneous therapy.

An analysis of the English literature was carried out. To search the scientific literature, we used the following electronic databases: Wiley Online Library, PubMed, COBISS. SI and Google Scholar web browser. We used keywords related to the fields of oncology, subcutaneous therapy, systemic therapy, and leadership in a healthcare organization. To combine key phrases in databases, we used Boolean operators (AND/IN).

We used the following inclusion and exclusion criteria: English language, publicly available material relevant to the set purpose and goal, and sources that were not older than 10 years. With the above criteria, we retrieved a definitive set of hits in the literature review. The results of the search for results by individual databases are presented in the table of results. The search was conducted in compliance with PRISMA guidelines, although this review was not registered with PROSPERO or any other registry.

## 3. Results

**Figure 1:**
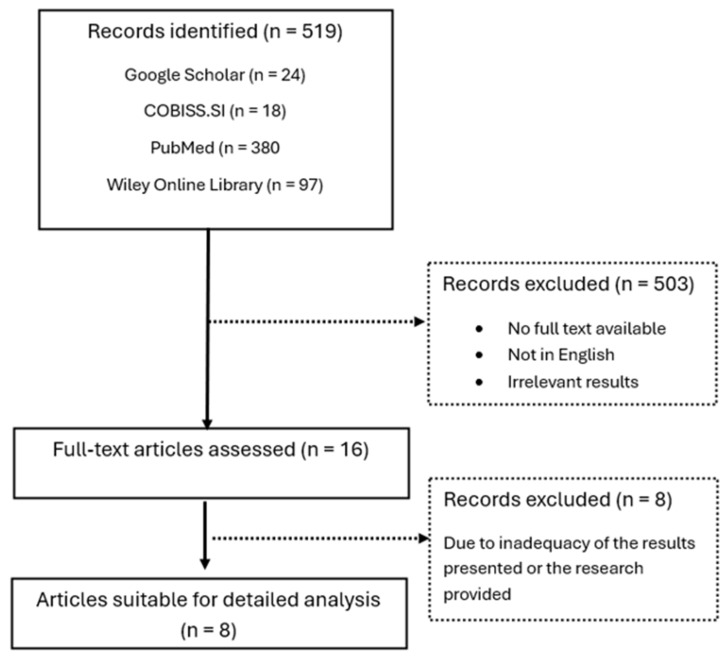
PRISMA diagram illustrating identification, screening, eligibility assessment, and final inclusion of studies.

The PRISMA diagram in Figure 1 and Table 1 illustrate the flow of studies through each stage of this review process, including identification, screening, eligibility assessment, and final inclusion. This review was conducted systematically by applying predefined criteria, removing duplicates, screening titles and abstracts, and evaluating full texts to ensure only relevant and high-quality studies were included. PRIMA checklist is available in the Supplementary Table S1. Eight studies were included in the qualitative synthesis. A formal risk-of-bias assessment was not performed, since the included studies were evaluated descriptively based on study design, sample size, and reported outcomes. Because we used a meta-synthesis approach and included primarily phase I–III clinical trials and one observational analysis, the risk of bias was considered narratively rather than through a structured tool. Given the qualitative nature of the synthesis and the heterogeneity of study designs, populations, and outcomes, no statistical assessment of heterogeneity or sensitivity analysis was conducted. Variability across studies was instead explored narratively by comparing pharmacokinetic, safety, and organizational outcomes. Certainty of evidence was not graded using GRADE or another formal framework. However, the overall evidence base consisted of multiple multicenter prospective trials, which generally provide moderate to high certainty for clinical outcomes.

Across eight multicenter prospective studies and one observational analysis conducted between 2024 and 2025, subcutaneous (SC) formulations of immune checkpoint inhibitors and targeted therapies consistently demonstrated pharmacokinetic comparability to parenteral (IV) administration, favorable safety and tolerability profiles, and meaningful advantages in patient experience and healthcare system efficiency, as shown in Table 2.

In two prospective studies evaluating pembrolizumab, SC administration showed strong pharmacokinetic alignment with IV dosing [4,6]. In a cohort of 140 patients with advanced solid tumors, SC pembrolizumab achieved pharmacokinetic exposure, bioavailability, and absorption rates comparable to the iv formulation at the selected dose [6]. These findings are reinforced by an analysis of 377 patients with NSCLC across multiple countries that reported that the overall exposure and trough concentrations with SC pembrolizumab supported its use across all indications previously treated with IV therapy [4].

Atezolizumab SC similarly showed favorable outcomes. In a study of 350 patients from the United States and Brazil, researchers found that SC atezolizumab maintained a safety profile consistent with IV administration while demonstrating improved tolerability [5]. Complementing these clinical findings, other researchers evaluated 179 patients and 46 healthcare workers across 14 European countries and observed high satisfaction with SC administration. Notably, the preparation time for the SC atezolizumab was approximately three times faster than for the IV formulation, highlighting operational efficiencies [11].

Nivolumab SC also demonstrated clinical and practical advantages [7]. In a study of 103 patients with advanced solid tumors, SC nivolumab exhibited an acceptable safety profile, enabled rapid administration, and was preferred by a greater proportion of patients compared with IV nivolumab [7].

Two large studies assessed SC amivantamab in patients with advanced EGFR-positive NSCLC [9,12]. In a cohort of 418 patients, SC amivantamab achieved noninferior pharmacokinetics and objective response rates relative to IV therapy, with signals suggesting longer response duration, progression-free survival, and overall survival [12]. Furthermore, in 416 patients, SC administration simplified and shortened treatment delivery, reduced resource utilization, enhanced the overall treatment experience, and was preferred by patients [9].

Beyond clinical outcomes, organizational benefits associated with SC therapy were documented [10]. In an observational analysis of 10 oncology clinics in Germany, it was found that the introduction of SC therapies reduced staff workload and increased patient throughput, underscoring the broader system-level advantages of transitioning from IV to SC administration.

This finding can also be easily calculated from the official durations of therapy administration shown in Table 3.

Across all evaluated agents, SC administration substantially reduced treatment time compared with IV infusion while maintaining established dosing intervals appropriate for each drug. For all immune checkpoint therapies, the required initial infusion is given over 60 min, followed by 30 min infusions for all subsequent doses. In contrast, the SC formulation can be administered in approximately 1–7 min, representing a marked reduction in chair time [13,14,15].

The most pronounced difference was observed with amivantamab. Parenteral administration involves prolonged infusion times, especially in the first cycle, ranging from 120 to 360 min, excluding the time for required premedication given with antihistamine, antipyretic and glucocorticoid drugs. By contrast, SC amivantamab can be administered in around 5 min for all doses, representing a transformative reduction in treatment duration by 24–70 fold [16].

Overall, SC formulations consistently shortened administration time from hours to minutes across all evaluated therapies, highlighting a substantial potential benefit for patients, infusion centers, and healthcare systems.

## 4. Discussion

The results of the literature review suggest that switching from parenteral (IV) to subcutaneous (SC) administration in patients with cancer is a clinically equally effective, safe, and more patient-friendly approach. Filip et al. [4] found that SC therapy maintains the same efficacy as IV, while significantly shortening application times, which has a positive effect on patients’ quality of life. Similar results were reported by Burotto et al. [5] who highlighted that the safety profile of the SC application is similar to that of the IV application, while the tolerability by patients is better, with fewer reactions and side effects.

Moeller et al. [10] conclude that SC application allows for better access to therapy as patients spend less time in the hospital, which also indirectly reduces the burden on oncology outpatient applications. The overall results confirm that SC therapy is not just a technical change but an improvement in terms of efficacy, safety and patient experience. The findings are consistent with those of a report by the European Society of Medical Oncology. Hendriks et al. [17] state that SC therapy improves the patient experience and reduces the need for long-term hospitalization.

The organizational impact of the transition to SC application is consistently recognized in the literature as one of the key factors motivating the introduction of changes. Moeller et al. [10] and Cappuzzo et al. [11] report that patient treatment time has been reduced by 40–60% with the introduction of the SC application, which translates into greater dispensary throughput, shorter wait times, and better staffing allocation. In the IMscin002 study, which included 179 patients and 46 healthcare professionals from 14 countries, patients and staff expressed a distinct preference for the SC administration of atezolizumab over the IV form, mainly due to shorter treatment time, greater comfort and lower workload of work processes [5,11]. In addition, it also proved to be psychologically advantageous for patients to spend as little time as possible in the day-hospital environment. Healthcare professionals estimate that the SC application allows more time flexibility, is less physically straining, and increases the time available to fully treat patients [11]. Digital transformation is also reshaping oncology workflows. A recent systematic review shows that digital tools in oncology improve treatment monitoring, reduce the risk of medication administration errors, and enhance communication among healthcare professionals [18].

The patient-reported outcomes (PROs) from recent multicenter studies consistently demonstrate that subcutaneous administration improves the overall treatment experience for patients with advanced NSCLC. Across trials evaluating SC pembrolizumab, atezolizumab, nivolumab, and amivantamab, patients reported greater comfort, reduced anxiety associated with venous access, and a strong preference for shorter treatment visits. In the IMscin002 study, more than 80% of patients preferred SC atezolizumab over IV infusion, citing reduced time in the clinic, less physical discomfort, and improved convenience in daily life. Similar findings were observed in studies of SC nivolumab and SC amivantamab, where patients highlighted the value of avoiding prolonged infusion sessions and the psychological benefit of spending less time in a hospital environment. Importantly, these improvements in patient experience occurred without compromising safety or efficacy. Collectively, PRO data indicate that SC administration not only maintains clinical outcomes but also enhances quality of life by reducing treatment burden and aligning therapy with patient-centered care principles.

As reported previously, the transition to the SC form of oncological therapy significantly reduces staff burden and is time-sparing for patients, as was the case with HER-2-positive breast cancer patients, where active treatment time and clinic visit duration were reduced by up to 96% and 53%, respectively. The total time spent in the chemotherapy suite decreased by 332 h over eight months, highlighting opportunities for resource reallocation. Nurses and pharmacists reported lower task complexity and greater workflow satisfaction, particularly during monotherapy. Therefore, SC administration has been well-established for patients who are HER-2-positive with applications fixed-dose combination of subcutaneous trastuzumab and pertuzumab. These data provide a practical precedent showing that SC delivery can reliably enhance quality of life, supporting similar expectations for SC immunotherapy and targeted therapy in lung cancer [19].

Therefore, we found that the transition to SC therapy in terms of organization reduces the burden on the health system and improves the fluidity, quality of care and economy of the provision of health services. The introduction of SC therapy represents an innovative change that requires strategic leadership, appropriate communication and employee involvement. Cummings et al. highlight that modern leadership styles, such as transformational and authentic leadership, drive innovation and increase employee engagement and satisfaction in healthcare teams. Leaders who invest in the development of employee competencies and involve them in decision-making increase the readiness of staff for change [20]. Therefore, we recommend a few workflow modifications, including streamlined patient flow, the reduced need for infusion chair occupancy, simplified preparation procedures, and the reallocation of nursing time toward complex care tasks. These adjustments support efficient and sustainable integration of SC administration into routine oncology practice.

From an economy perspective, the transition from intravenous to subcutaneous administration does not increase drug acquisition costs, as list prices of SC formulations are aligned with those of the higher-dose IV formulations across Germany, Italy, France, and Slovenia. The primary financial advantage of SC therapy therefore arises from reduced chair time, shorter preparation and administration workflows, and lower staffing requirements. These operational efficiencies translate into improved patient throughput, decreased burden on infusion units, and more flexible allocation of nursing resources. In several studies, SC administration reduced treatment time by 40–70%, suggesting that the overall cost of care is substantially lower despite similar per-dose pricing. Consequently, the financial value of SC therapy is driven not by the price of the drug but by system-level savings and improved utilization of healthcare resources [21,22,23,24].

This review was limited by the absence of a preregistered protocol, lack of a structured risk-of-bias assessment, and potential publication bias due to the reliance on the published literature. The search strategies did not include the grey literature or conference abstracts, which may have excluded emerging evidence.

Nevertheless, we look forward to researchers conducting strong real-world studies to determine whether subcutaneous formulations truly deliver the expected time savings and benefits for all patients in our care. Until then, this type of therapy delivery is currently the best option available for both patient and healthcare staff satisfaction [25].

## 5. Conclusions

The results of this literature review confirmed that the SC application of therapy is an important innovation in oncology treatment, which provides benefits to patients, healthcare professionals, and the organization as a whole. The introduction of SC oncological therapy is an important step towards the sustainable development of health services. It enables the better use of time, enhances patient satisfaction, reduces the burden on healthcare professionals, and fosters an innovative culture in healthcare organizations. For successful implementation, it is necessary for organizations to be aware of the importance of education, communication, and strategic change management. The SC application of oncological therapy represents an important innovation in modern oncology, with its success depending on health professionals, their leaders, and their readiness for change.

## Figures and Tables

**Table 1:** The results of the literature review.

Database	Keywords	Number of Hits	Selected Hits to Review in Full Text
Google Scholar	Transition from parenteral to subcutaneous application of oncological therapy	24	3
COBISS.SI	Subcutaneous oncology therapy AND lung cancer	18	1
PubMed	Subcutaneous oncology therapy AND lung cancer	380	8
Wiley Online Library	Oncology innovation AND change management	97	4
TOTAL		519	16

**Table 2:** Overview of the main results from the trials comparing parenteral and subcutaneous formulations of lung cancer therapies.

Author	Year	Exploratory Design	Sample Size	Key Takeaways
Cohen et al. [6]	2025	Multicenter prospective research	140 patients with advanced solid tumors	Pembrolizumab SC has similar pharmacokinetic exposure, bioavailability, and absorption rate to IV formulation at chosen dose level
Felip et al. [4]	2025	Multicenter prospective research	377 patients with NSCLC, multinational	Overall exposure and trough concentrations of SC application of pembrolizumab support SC as a treatment option in all indications where IV therapy was previously used
Burotto et al. [5]	2024	Multicenter prospective research	350 patients, USA and Brazil	Atezolizumab SC shows similar safety and better tolerability in patients
Lonardi et al. [7]	2025	Multicenter prospective research	103 patients with advanced solid tumors	Nivolumab SC has an acceptable safety profile, allows for rapid administration, and is preferred by more patients than nivolumab IV
Leigh et al. [8]	2024	Multicenter prospective research	418 patients with advanced EGFR-positive NSCLC	Amivantamab in SC formulation had noninferior pharmacokinetics and objective response rates, with potentially longer response duration, progression-free survival, and overall survival compared with amivantamab IV
Alexander et al. [9]	2025	Multicenter prospective research	416 patients with advanced EGFR-positive NSCLC	Amivantamab SC simplifies and shortens administration time, reduces resource utilization, enhances treatment experience and is a preferred option for patients
Moeller et al. [10]	2024	Observational analysis of organizational effects	10 oncology clinics, Germany	Introduction of SC therapy reduces workload on staff and increases patient throughput
Cappuzzo et al. [11]	2025	Prospective multicenter research	179 patients and 46 healthcare workers, 14 countries (EU)	Medical staff and patients express high satisfaction with SC application; preparation time of atezolizumab SC approximately three times faster

**Table 3:** Treatment options that are available in both parenteral and subcutaneous formulations for the treatment of advanced non-small-cell lung cancer and their time of application in minutes; IV—intravenous; SC—subcutaneous; Q2W—every two weeks; Q3W—every 3 weeks; Q4W—every 4 weeks.

Drug Name	Dose and Interval IV	Application Time IV	Dose and Interval SC	Application Time SC
Atezolizumab	1200 mg IV Q3W 1680 mg IV Q4W	First 60 min, then 30 min (all doses)	1875 mg SC Q3W -	7 min
Nivolumab	240 mg IV Q2W 360 mg IV Q3W 480 mg IV Q4W	First 30 min, then 60 min (all doses)	600 mg SC Q2W - 1200 mg SC Q4W	3–5 min - 3–5 min
Pembrolizumab	200 mg IV Q3W 400 mg IV Q6W	First 30 min, then 60 min (all doses)	395 mg SC Q3W 790 mg SC Q6W	>1 min >2 min
Amivantamab	1050 mg/1400 mg IV weekly for 4 weeks, then Q2W 1750 mg/2100 mg IV weekly for 4 weeks, then Q3W	C1D1 240 min C1D2 240–360 min C1D8 180–240 min C1D15 120–180 min From C2 to 120 min	1600 mg/2240 mg SC weekly for 4 weeks, then Q2W -	>5 min (all doses)

## Data Availability

Not applicable.

## Supplementary Materials

The following supporting information can be downloaded at: https://www.mdpi.com/article/10.3390/curroncol33060307/s1, Table S1: PRISMA checklist.

## References

[1] WHO (2025). A Global Health Strategy for 2025–2028.

[2] Indicators O. (2023). Health at a Glance 2023.

[3] George S., Bourlon M.T., Overman M.J., Dixon M., Shelley K., Markus K.J., Kewley R.M., Pope S.I., Albig L. (2025). Systematic Literature Review of Intravenous versus Subcutaneous Administration of Oncology Therapies: A Clinical, Economic and Patient Perspective. Cancer Treat. Rev..

[4] Felip E., Rojas C.I., Schenker M., Kowalski D.M., Casarini I.A., Csöszi T., Şendur M.A.N., Martins J., Calles Blanco A., Wang C.C. (2025). Subcutaneous versus Intravenous Pembrolizumab, in Combination with Chemotherapy, for Treatment of Metastatic Non-Small-Cell Lung Cancer: The Phase III 3475A-D77 Trial. Ann. Oncol..

[5] Burotto M., Zvirbule Z., Mochalova A., Runglodvatana Y., Herraez-Baranda L., Liu S.N., Chan P., Shearer-Kang E., Liu X., Tosti N. (2023). IMscin001 Part 2: A Randomised Phase III, Open-Label, Multicentre Study Examining the Pharmacokinetics, Efficacy, Immunogenicity, and Safety of Atezolizumab Subcutaneous versus Intravenous Administration in Previously Treated Locally Advanced or Metastati. Ann. Oncol..

[6] Cohen G.L., Coetzee C., Walton C.A., Chul B., Mcadam G., Rojas C.I., Medina L., Papai Z., Chan S.W., Rapoport B.L. (2026). Pharmacokinetics and Bioavailability of Pembrolizumab with Berahyaluronidase Alfa for Subcutaneous Administration in Participants with Advanced or Metastatic Solid Tumors: The Phase 1 Study 3475A-C18. Eur. J. Cancer.

[7] Lonardi S., Ługowska I., Donnell A.O., Jackson C., Latten- L.M., North R., Bahleda R., Garrido M., Santoro A., Chacon M.R. (2025). Pharmacokinetics and Safety of Subcutaneous Nivolumab: Results from the Phase I/II CheckMate 8KX Study. J. Immunother. Cancer.

[8] Leighl N.B., Akamatsu H., Lim S.M., Cheng Y., Minchom A.R., Marmarelis M.E., Sanborn R.E., Yang J.C., Liu B. (2024). Subcutaneous Amivantamab vs Intravenous Amivantamab, Both in Combination with Lazertinib, in Refractory EGFR-Mutated, Advanced Non-Small Cell Lung Cancer (NSCLC): Primary Results, Including Overall Survival (OS), from the Global, Phase 3, Randomized Controlled PALOMA-3 Trial. J. Clin. Oncol..

[9] Alexander M., Cheng Y., Lee S., Passaro A., Spira A.I., Chul B., Min S., Ohe Y., Nagrial A., Liang J. (2026). Subcutaneous versus Intravenous Amivantamab, Both in Combination with Lazertinib, in Refractory EGFR -Mutated Non-Small Cell Lung Cancer: Patient Satisfaction and Resource Utilization Results from the PALOMA-3 Study. Eur. J. Cancer.

[10] Moeller J., Green M.D., Ramnath N. (2024). Pros and Cons of Subcutaneous (SC) versus Intravenous (IV) Administration of Immune Checkpoint Inhibitors in Non-Small Cell Lung Cancer. Transl. Lung Cancer Res..

[11] Cappuzzo F., Zvirbule Z., Korbenfeld E., Yovanna A., Sanchez C., Bustillos A., Liu X., Majem M. (2025). Primary Results from IMscin002: A Study to Evaluate Patient Preferences and Perceptions of Health Care Professionals for Atezolizumab Subcutaneous Versus Intravenous for the Treatment of NSCLC. JTO Clin. Res. Rep..

[12] Leighl N.B., Akamatsu H., Lim S.M., Cheng Y., Minchom A.R. (2024). Subcutaneous Versus Intravenous Amivantamab, Both in Combination with Lazertinib, in Refractory Epidermal Growth Factor Receptor—Mutated Non—Small Cell Lung Cancer: Primary Results from the Phase III PALOMA-3 Study. J. Clin. Oncol..

[13] Tecentriq Smpc. https://ec.europa.eu/health/documents/community-register/2018/20180404140503/anx_140503_en.pdf.

[14] Opdivo Smpc. 1–292. https://www.ema.europa.eu/en/documents/product-information/opdivo-epar-product-information_en.pdf.

[15] Keytruda Smpc. 1–159. https://www.ema.europa.eu/en/documents/product-information/keytruda-epar-product-information_en.pdf.

[16] Rybrevant Smpc. 1–80. https://www.ema.europa.eu/en/documents/product-information/rybrevant-epar-product-information_en.pdf.

[17] Hendriks L.E., Kerr K.M., Menis J., Mok T.S., Nestle U., Passaro A., Peters S., Planchard D., Smit E.F., Solomon B.J. (2023). Oncogene-Addicted Metastatic Non-Small-Cell Lung Cancer: ESMO Clinical Practice Guideline for Diagnosis, Treatment and Follow-Up☆. Ann. Oncol..

[18] Tuominen L., Leino H., Jenna K., Daniela P., Carme C., Leeni C. (2024). Interactive Digital Tools to Support Empowerment of People with Cancer: A Systematic Literature Review. Support. Care Cancer.

[19] Oshi M., Nakayama T., Ota I., Sasamoto M., Yamada A., Narui K. (2026). Multidimensional Impact of Fixed-Dose Subcutaneous Trastuzumab- Pertuzumab on Oncology Workflow and Patient Time Burden in a Real-World Study. Breast Cancer.

[20] Kapra O., Asna N., Amoyal M., Bashkin O., Dopelt K. (2023). The Oncology Clinical Nurse Specialist: A Rapid Review of Implementation Models and Barriers around the World. Curr. Oncol..

[21] Germany—Lauer-Taxe Lauer-Taxe. Arzneimitteldatenbank für Deutschland. https://www.cgm.com/deu_de/loesungen/apotheke.html.

[22] Italy—AIFA (Agenzia Italiana del Farmaco) AIFA Banca Dati Farmaci—Prontuario Farmaceutico Nazionale. https://medicinali.aifa.gov.it/en/#/en/.

[23] France—Base de Données Publique des Médicaments (BDPM). https://base-donnees-publique.medicaments.gouv.fr.

[24] Slovenia—ZZZS Centralna Baza Zdravil (CBZ) Zavod za Zdravstveno Zavarovanje Slovenije Centralna Baza Zdravil. https://partner.zzzs.si/.

[25] Sentana-lledo D., Gupta A. (2026). Subcutaneous Immunotherapy: It Is Time for Real-World Data. JCO Oncol. Pract..

