# Supplementary material for: Transition from Parenteral to Subcutaneous Application of Systemic Oncological Therapy for Treating Non-Small-Cell Lung Cancer
Source: Curr Oncol. 2026 May 25;33(6):307. doi: 10.3390/curroncol33060307 (PMC13297852; doi:10.3390/curroncol33060307)
Supplement: Supplementary file 1 [file curroncol-33-00307-s001.zip › curroncol-4247110-supplementary.pdf]

Transition from parenteral to subcutaneous application of systemic oncological therapy for the treatment of non-small cell lung cancer

Anela Muratovic, Urska Janzic

Supplementary Table S1

Item	Checklist Item	Reported?	Location in Document
1	Identify as a systematic review	Reported	Title + Methods
2	Structured abstract	Reported	Abstract
3	Rationale	Reported	Methods
4	Objectives	Reported	Methods
5	Eligibility criteria	Reported	Methods
6	Information sources	Reported	Databases listed
7	Search strategy	Partially	Keywords given, not full strings
8	Selection process	Reported	PRISMA flow described
9	Data collection process	Reported	Methods
10a	Outcomes	Reported	Results
10b	Other variables	Reported	Results
11	Risk of bias assessment	Reported	Results
12	Effect measures	Reported	Results
13a	Synthesis methods	Reported	Results
13b	Heterogeneity	Reported	Results
13c	Sensitivity analyses	Reported	Results
14	Reporting bias assessment	Reported	Results
15	Certainty assessment	Not reported	Results
16a	Study selection	✓ Reported	Results

16b	Excluded studies with reasons	✓ Reported	Results and Figure 1
17	Study characteristics	Reported (Table 2)	Results
18	Risk of bias in studies	Not reported	-
19	Individual study results	Reported	Results
20a	Synthesis	Reported	Discussion
20b	Statistical synthesis	Not applicable	-
21	Reporting bias	Partially	Discussion
22	Certainty of evidence	Not applicable	-
23a	Summary	Reported	Discussion
23b	Limitations of evidence	Partially	Discussion
23c	Limitations of review process	Reported	Discussion
23d	Implications	Reported	Submission process
24	Registration	Reported	Submission process
25	Protocol	Reported	Submission process
26	Funding	Reported	Submission process
27	Competing interests	Reported	Submission process
28	Data availability	NA Not applicable	Submission process
